# Novel cardiac cell subpopulations: Pnmt-derived cardiomyocytes

**DOI:** 10.1098/rsob.200095

**Published:** 2020-08-19

**Authors:** Alexander Grassam-Rowe, Xianghong Ou, Ming Lei

**Affiliations:** 1 Department of Pharmacology, University of Oxford, Oxford OX1 3QT, UK; 2 Key Laboratory of Medical Electrophysiology of the Ministry of Education and Institute of Cardiovascular Research, Southwest Medical University, Luzhou 6400, People's Republic of China

**Keywords:** Pnmt-derived cardiomyocytes, PdCMs, phenylethanolamine *N*-methyl transferase, Pnmt, cardiac adrenergic signalling, cardiac cellular subpopulations

## Abstract

Diversity among highly specialized cells underlies the fundamental biology of complex multi-cellular organisms. One of the essential scientific questions in cardiac biology has been to define subpopulations within the heart. The heart parenchyma comprises specialized cardiomyocytes (CMs). CMs have been canonically classified into a few phenotypically diverse subpopulations largely based on their function and anatomic localization. However, there is growing evidence that CM subpopulations are in fact numerous, with a diversity of genetic origin and putatively different roles in physiology and pathophysiology. In this chapter, we introduce a recently discovered CM subpopulation: phenylethanolamine-*N*-methyl transferase (Pnmt)-derived cardiomyocytes (PdCMs). We discuss: (i) canonical classifications of CM subpopulations; (ii) discovery of PdCMs; (iii) Pnmt and the role of catecholamines in the heart; similarities and dissimilarities of PdCMs and canonical CMs; and (iv) putative functions of PdCMs in both physiological and pathological states and future directions, such as in intra-cardiac adrenergic signalling.

## 1. Introduction

Complex multi-cellular organisms arise from the interactions of specialized organ systems. These organ systems arise from specialized groups of cells in the tissues that constitute various distinct organs. The study of how interactions between differentiated cell types ultimately give to rise to complex life as we know it remains under intense philosophical interrogation in current biological scientific practice. Cardiac biology concerns the study of the heart, including the study of its interacting cell types. The heart's major physiological function is to act as a pump in the cardiovascular system to provide a continuous circulation of blood throughout the body. The heart achieves this by the coordinated contraction and relaxation of billions of cardiomyocytes (CMs) around a fibrous skeleton. The coordination is achieved through the propagation of an electrical signal from the right atrium of the heart, to the left atrium and the ventricles, resulting in excitation-contraction coupling (ECC) across the myocardium in a specific temporal sequence to maximize the efficiency of the heart as a pump. This contraction, and the appropriate generation and propagation of the coordinating signal are mediated by CMs. However, those associated with the generation and propagation of the electric signal are more specifically differentiated than working CMs. The phenotypic difference between these two populations of CMs facilitates the normal rhythmic pumping of the heart. In recent years, it has been recognized that the heart is in fact more heterogeneous in cellular phenotypes than canonically thought. Not only has our understanding of the non-cardiomyocytes populations in the heart increased, but it is now evident that CM heterogeneity extends beyond the early canonical atrial versus ventricular [1,2], and non-working versus working differences [2,3]. Our group has discovered a novel CM, through lineage cell fate tracing – phenylethanolamine-*N*-methyl transferase (Pnmt)-derived cardiomyocytes (PdCMs) [4,5]. PdCMs have a different developmental history than most CMs, and are descended from cells that have at some point during development stages expressed Pnmt [4–6]. PdCMs have a selective mostly left-sided anatomical distribution in the adult heart [6,7]. A small percentage of PdCMs actively express Pnmt in the adult heart, although the functional corollary of this, if any, is currently uncertain [5–7]. In this chapter, we outline: the current classification of CMs, the history of PdCMs, the history of Pnmt, known similarities and dissimilarities of PdCMs, and future directions for the field.

## 2. Pnmt^+^-derived cardiomyocytes

### 2.1. Current classifications of cardiomyocytes

**Figure 1.**
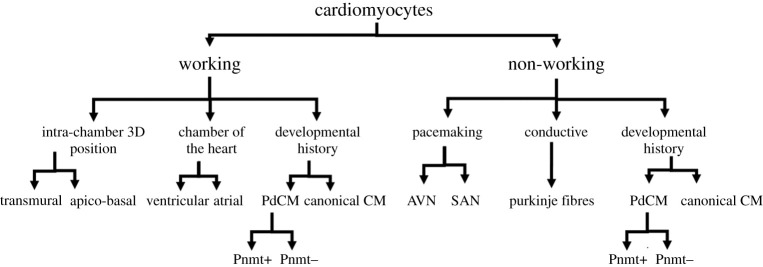
Cardiomyocytes can be classified by several anatomical, structural and functional characteristics. Each generation describes a type of variation for consideration in classification, but is non-exhaustive. CM, cardiomyocyte; PdCM, Pnmt-derived cardiomyocyte; AVN, atrioventricular node; SAN, sinoatrial node.

It has been recognized for a long time that the heart has intrinsic cellular heterogeneity in terms of cell types and their electrophysiological properties, despite its apparently simple and unitary pumping function. Figure 1 demonstrates some aspects of variation in classification. Early discussion of heterogeneity focused mostly on the differences between the pacemaking and conductive elements of the heart, namely the nodal tissue and His-Purkinje system, versus that of the more contractile myocardium proper. Initially, the main focus was histological differences that identified the specialized conducting and pacemaker cells as being specialized compared to working CMs [3,8,9]. This study then grew to identifying the intrinsic electrophysiological differences, such as in action potential morphology, and the lack of stable resting membrane potential in nodal pacemaker cells compared to the resting membrane potential of working CMs [10].

Over time, with further study and improved techniques, it became apparent that the cellular heterogeneity of the heart expanded even further within these canonical pacemaking, conductive and working CM classifications. Within the pacemaking/conductive system for example, even today there is controversy surrounding classification of nodal cell heterogeneity in terms of morphology and electrophysiology [10]. Within working CMs, it is now recognized that CMs differ across the heart's chambers, transmurally within a given chamber [11], and even along the apico-basal axis of the heart [12]. Such differences in working CMs phenotypes include contractility, electrophysiology and pharmacology [11,12].

There is also heterogeneity in the additional roles of CMs beyond simply pacemaking, conductive or working. Although the endocrine potential of CMs is well recognized, with the natriuretic peptides being secreted by atrial and ventricular working CMs [13]; recent studies have identified more local hormonal signalling, with certain CMs being identified that appear to possess the complete synthetic machinery for the production, storage and synthesis of acetylcholine (ACh) [14], and that appear to actively secrete ACh under certain conditions [15,16]. However, the precise functional role of these ACh-secreting CMs remains under investigation [16].

In summary, CMs are a heterogeneous population. CMs can be classified broadly as non-working and working. The non-working CMs can be further classified as pacemaking and conductive fibres. Working CMs themselves can similarly be classified as atrial or ventricular. However, within these subpopulations for both non-working and working CMs, there exist further subpopulations of phenotypically distinct CMs. The past decade, with the expansion of novel interrogative techniques has further identified ever-increasingly discrete classifications of CMs.

### 2.2. Discovery of Pnmt-derived cardiomyocytes

Recent work has identified a previously unknown subpopulation of CMs: PdCMs. Work by Dr Ebert, developed a mouse model in which the native Pnmt locus was modified by insertion of a gene encoding Cre-recombinase as an open reading frame at the 5′ exon 1 of the gene encoding Pnmt [4]. The Pnmt-Cre mouse was then crossed with a Rosa26R reporter strain. The R26R strain contains loxP sites that inhibit a reporter gene (LacZ) transcription. The crossed Pnmt-Cre x Rosa26R mice were developed such that if a cell, at any point in its developmental history, activates the transcription of Pnmt, it would activate transcription of the Cre-recombinase, which would lead to permanent expression of the reporter LacZ (encoding Beta-galactosidase) in that cell and any descendants. It was observed in mouse embryos as early at E8.5, that LacZ+ cells were present in specific areas of the developing heart in the Pnmt-Cre/Rosa26R mice. At E10.5, the distribution of this staining was even more extensive in the heart. Sagittal sectioning of the developing E10.5 mouse hearts into 20 μm sections and staining with XGAL, which detects the gene product of LacZ, identified that this expression of LacZ was distributed across the atria and ventricles, but with strongest staining around junctional regions. The pattern of staining was mostly localized within the myocardium, prompting investigation of the cellular source of the staining. Immunofluorescent staining for sarcomeric alpha-actinin, a muscle-specific marker, displayed overlap with both immunofluorescent staining for Pnmt and with histochemical staining for XGAL [4]. Previously, Pnmt in the heart was known as a marker of ‘intrinsic cardiac adrenergic’ (ICA) cells, which display a neuroendocrine-like morphology and phenotype [17,18]. However, the overlap of Pnmt, with LacZ, and the canonical muscle marker alpha-actinin, suggested that the cell type in question did not fit known features of the ICA cells, which are not known to express sarcomeric alpha-actinin. However, CMs have a strong expression profile of sarcomeric alpha-actinin, suggesting that some CMs were either expressing Pnmt currently, or the XGAL staining overlap indicating that their progenitor cells had at some point expressed Pnmt [4]. Further investigation in E15.5 mouse embryos, which have a more adult heart morphologically, suggested that this myocardial LacZ+ XGAL staining persisted. Additionally, in these E15.5 hearts, there was extensive overlap of HCN4 immunofluorescence and XGAL staining in the sinoatrial node (SAN). HCN4 is a selective marker of pacemaker CMs within the SAN. These experiments were the first to identify this novel developmental lineage for CMs from progenitors that had, at least transiently, expressed Pnmt [4].

### 2.3. Unique distribution of Pnmt-derived cardiomyocytes

Further work identified that these XGAL+ cells appear to have a specific distribution that differs between the developing and adult heart. In the developing heart at E10.5, XGAL staining was positive for LacZ expression sporadically within all chambers of the heart. However, the sinoatrial junction, atrioventricular and conoventricular junctions had particularly dense staining [4,19]. Furthermore, at E15.5, the SAN and atrioventricular node (AVN) had extensive staining of XGAL, with approximately half of HCN4+, presumed pacemaker, cells in the SAN also co-staining positive with XGAL. However, by E15.5 there was extensive staining for XGAL throughout the myocardium, with particularly strong staining in the interventricular septum, which extended into both ventricles and towards the apex. Within the ventricular walls, there was some sparse XGAL staining, which focused mostly, but not exclusively, near the endocardial surface. To confirm the muscular nature of these XGAL+ cells, concomitant immunofluorescence for sarcomeric alpha-actinin was used to confirm that many of the XGAL+ cells had overlapping signal for sarcomeric alpha-actinin, and were not simply XGAL+ ICA cells. Thus, within the developing heart, there is clearly a dynamic distribution of these XGAL+ cells arising from the initial population that appears to be focused mostly within the developing pacemaker/conduction system, before expanding out to the remainder of the myocardium [4,19].

However, it was then elucidated that the distribution of XGAL+ cells was much more specifically left-sided than in the developing heart. Using the same cross of Pnmt-Cre-Rosa26LacZ mice, but isolating the hearts at 8–10 weeks of age demonstrated that XGAL staining was much more selective to the left atrium and left ventricle [6] The left atrial myocardium had fairly extensive staining with XGAL. However, within the left ventricle, the distribution was much more specific. Using serial sections of 14**–**20 μm, it was demonstrated that while the apex displayed strong staining for XGAL, more dorsal sections displayed stronger XGAL staining more medially, and stronger within the interior of the heart than around the periphery. Upon reaching the most dorsal sections, the base of the heart had more extensive staining throughout the left ventricle. The right-sided distribution was much less extensive, and tended to be small clusters of XGAL+ cells in the interventricular septum and the right ventricular wall. The left-sided distribution was so stark that quantitative pixel by pixel analysis of the sections, following delineation into left and right divisions, suggested that an average of 89% of XGAL+ cells were present in the left side of the heart when analysing five random slices per heart, across three hearts [6]. High magnification phase-contrast, light microscopy and co-staining for XGAL and sarcomeric alpha-actinin identified that many of the XGAL+ cells in the adult heart were CM-like [6].

**Figure 2.**
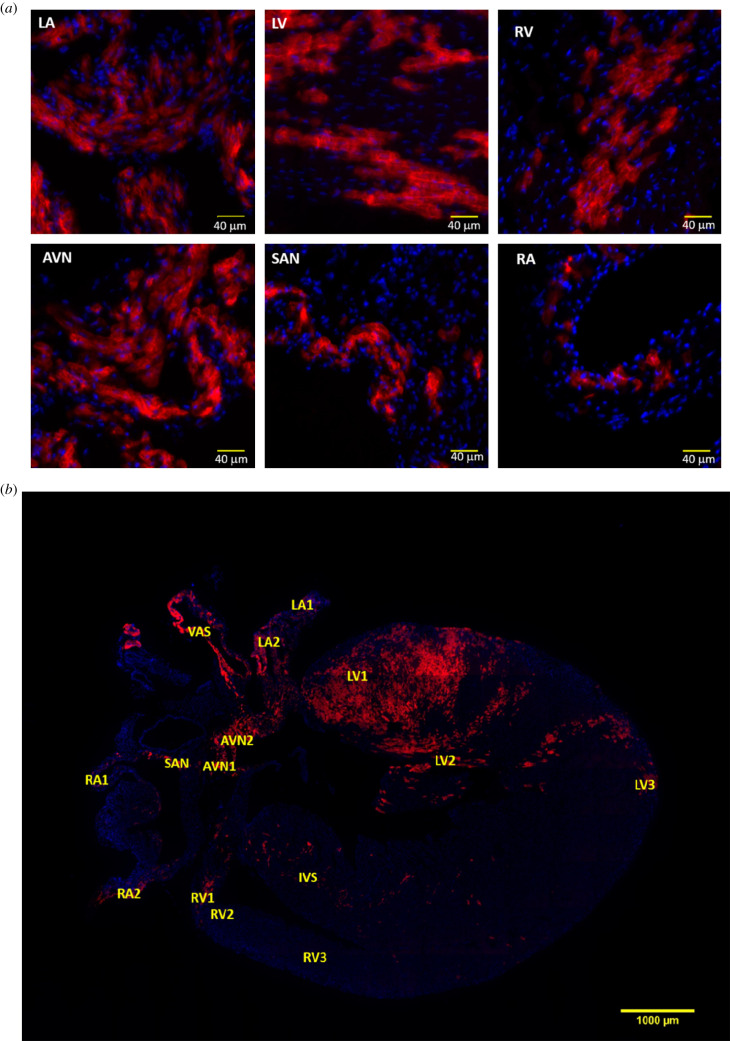
The distribution of PdCMs in the adult mouse heart is predominantly left-sided. Representative images of the coronal section and selected regions from the section of an adult ChR2/tdTomato mouse heart showing fluorescence and morphology of the ChR2/tdTomato positive cells. (*a*) A representative coronal section from an adult ChR2/tdTomato mouse heart; (*b*) inserts of zoom-in views showing tdTomato fluorescence in different regions of the heart; the labelling of the inserts indicates the corresponding locations as marked in (*a*). AVN, atrioventricular node; LA, left atrium; LV, left ventricle; SAN, sinoatrial node; RA, right atrium, RV, right ventricle. Figure reproduced from [7].

This left-side preferential distribution and electrophysiological properties of PdCMs in the adult heart was confirmed, to our knowledge, for the first time by our group as seen in figure 2 [5,7]. Thus, our group confirmed the distribution and electrical activity of PdCMs in an adapted mouse optogenetic model. We developed a novel mouse strain by crossing Pnmt-Cre mice with Ai27D mice that expressed an improved channelrhodopsin-2(ChR2)/tdTomato fusion protein, following excision of a STOP sequence that was flanked with -loxp sequences. The fluorescence signal from tdTomato provided an excellent endogenous marker to identify PdCMs. Coronal sections of hearts agreed with earlier work [6] that the left atrium, left ventricle and interventricular septum were particularly rich in PdCMs [5]. In remarkable proximity to earlier quantitative approaches, an average of 86% of ChR2/tdTomato+ cells were on the left side of the heart. Moreover, this study now quantitatively reported the contribution of PdCMs to the overall number of CMs; PdCM estimates suggest that approximately 50% of the left atrium, and 21% of the left ventricle CMs are PdCMs [5]—in agreement with the extensive distribution of PdCMs in the left atrium in earlier studies [6], whereas in the right atrium PdCMs only accounted for 7% of CMs, and in the right ventricle only 2%. These estimates provide concrete quantitative evidence of the significance of PdCMs in terms of developmental heterogeneity in the heart. Moreover, in agreement with earlier studies, we reported a great deal of co-localization of HCN4+ cells with tdTomato+ cells in the AVN, but with less so in the SAN—suggesting that the AVN has a richer population of PdCMs than the SAN [5]. In addition, those tdTomato+ cells appear to have a more peripheral distribution within the SAN. We then used a wide-field deconvolution method of fluorescence microscopy with ChR2/tdTomato+ adult mouse hearts, to produce whole heart high-resolution images. Such wide-field but high resolution imaging enabled us to produce a reconstructed three-dimensional map of PdCMs globally across the heart, while maintaining resolution similar to confocal microscopy in terms of cellular details—and confirmed the distributions of the ChR2/tdTomato+ cells primarily within the left side and pacemaker and conduction systems of the heart [5,7].

In summary—PdCMs were identified through cell fate lineage tracing for cardiac expression of Pnmt in the developing heart [4]. Unexpectedly, it was elucidated that a significant proportion of cells with a history of Pnmt expression in their development was not confined to just ICA cells and neural crest-derived (NCD), but also included CM-like cells types [4,20]. Furthermore, these CM-like cell populations underwent dynamic transformations during cardiac development [4], but then came to occupy a mostly left-sided distribution in the adult heart [5–7], and contribute significantly to the total CM count in the left atrium and ventricle [5–7].

## 3. Phenylethanolamine-*N*-methyl transferase

Pnmt is an enzyme that catalyses the final step in the synthesis of adrenaline. Adrenaline is produced by the *N*-methylation of noradrenaline. Noradrenaline is, in turn, synthesized from dopamine, which originates from l-dopa — a derivative of the amino acid tyrosine. Pnmt was first identified by J. Axelrod in 1962 [21]. Axelrod deftly isolated and purified the enzyme from extracts of several organs from several species and identified the chemical products from related catecholamine substrates [21].

Pnmt expression and activity is restricted to certain tissues [22]. The adrenal glands are the prime site of Pnmt expression, across species [21,22]. However, the sum of extra-adrenal Pnmt activity is reported to be equal to that observed within the adrenals [22]. In adult rat tissue lysates, the strongest activity was observed in hypothalamic and atrial extracts, although Pnmt is also reported in other brain regions, and across other tissues such as the eyelid, in skeletal muscle, the kidney and the retina [22].

### 3.1. Phentolamine-*N*-methyl transferase in the heart

Pnmt expression in the heart is drawn into further questioning by the discovery of PdCMs. Even since Axelrod's first identification of Pnmt [21], it was observed that the heart possesses some intrinsic Pnmt activity. Axelrod observed this to be the case in extracts of rabbit and monkey heart, although it was much lower than that he observed in the adrenal glands of several species [21]. Later work identified that catecholamines in the heart were not produced solely by sympathetic neurons, with their well-known predominantly catecholaminergic signalling [23,24]. Ignarro & Shideman identified from chick embryonic cardiac tissue homogenates that different developmental stages were associated with a dynamic expression of the catecholamines l-dopa, dopamine, noradrenaline and adrenaline, and the expression of their respective synthetic enzymes in the heart and that this occurred prior to sympathetic innervation of the heart on the 6th day of incubation [23,25,26]. In fact, during the early stages of development in the chick embryonic heart, 85**–**95% of the catecholamines are adrenaline [25]. This was later confirmed in rats to the degree that at E10, heart lysate Pnmt mRNA was the major contributor to an associated whole embryo surge. The concomitant Pnmt activity in the rat heart at E10 was quantitatively similar to that observed in extracts from the adrenals later in development [27]. Furthermore, the heart also displays activity for catecholamine degradative enzymes—catechol-*O*-methyl transferase and monoamine oxidase prior to sympathetic innervation [23]. Additionally, Ignarro & Shideman identified that the uptake of noradrenaline persisted in non-innervated hearts, and that it was much less effected by reserpine and cocaine, which prevent NET-mediated reuptake and storage in sympathetic neurons—suggesting a non-neuronal source of noradrenaline uptake and storage in the heart [26]. Thus, even from early studies of catecholamines in the developing heart it became clear that there was some cellular source of both catecholamine production, degradation and storage that was not merely the sympathetic innervation.

Subsequent work has since confirmed the prevalence of catecholamines within the heart from non-neuronal sources, including in humans, during development [17,24,28]. These cells have been termed ICA cells [28]. These cells appear to have a widespread distribution across all four chambers of the developing heart, before coming to occupy a predominantly left-sided distribution in the adult heart [4]. At E10.5 in mice, neural crest cells also invade the heart [4], with different subpopulations contributing to different cell populations in the adult, such as sympathetic neurons [29], and parasympathetic neurons [29]. While these neural crest cells do express Pmnt, at least transiently, there is very limited evidence of any contribution to mature myocytes [30–32], and so are unlikely to contribute to PdCMs. Various studies suggested neural crest cell-like markers are expressed in the developing cardiac conduction system [30]. However, it appears that these markers are expressed even before neural crest invasion of the heart [32], suggesting that the markers' expression is merely coincidentally common in neural crest and the developing conduction system. Thus, ICAs in the developing heart are believed to contribute to the PdCMs we have observed, although this remains to be definitively proved. The totality of cellular sources of catecholamine synthesis enzymes remains an area of dispute and exploration, building on the significant corpus of literature surrounding the functional effects of catecholamines on the heart.

### 3.2. Role of catecholamines in regulating the adult heart

Catecholamines act within the adult heart to effect a response to acute and chronic stress. CMs express a cassette of adrenoreceptors, with beta-1 isoforms being the most prevalent on adult CMs [33]. While dopamine is known to exhibit effects in the heart [34], the majority of catecholaminergic signalling in the adult heart is mediated by noradrenaline released from sympathetic neurons [35], or adrenaline mostly circulating in the blood [36]. Catecholamines act, primarily through beta-1 adrenoreceptors, on working CMs to induce a positive inotropic [37], clinotropic [37] and lusotropic effect [38–40], while acting on non-working CMs to induce a positive chronotropic [41–43] and dromotropic effect [44]. This contributes to an acutely increased cardiac output in acute stress [45]. Furthermore, with chronic stress, catecholaminergic signalling contributes to the cellular hypertrophy and organ remodelling that ensues. However, it is currently unclear whether catecholamines contribute to physiological remodelling, such as in exercise [46–48]. However, the contribution of catecholaminergic signalling is well-established in more pathological remodelling such as during heart failure [47,49,50]. However, catecholaminergic signalling is implicated in increased risk of cardiac dysrhythmia [51,52]. Sudden cardiac death is significantly associated with acute adrenergic stresses precipitating cardiac dysrhythmia. Additionally, the cardiac remodelling associated with sustained catecholaminergic signalling can lead to maladaptive remodelling with disrupted cardiac anatomy and myocardial calcium handling further precipitating dysrhythmia risk [53,54]. Thus, in the adult heart catecholamine signalling has acute and chronic effects, which can both be either physiological or pathological.

### 3.3. Role of catecholamines in the developing heart

Catecholamines in the developing heart may be important for coordinating the electrical activity of the heart. Multiple groups have identified the association of catecholamine producing cells in close association with the developing pacemaker and conduction systems of the heart, both putatively ICAs [28] and NCDs [30] but starting at different stages of development. At E10.5 in rats, the primordial conduction system appears to have a strong expression of catecholaminergic biosynthetic enzymes, including around the site of the future SAN and AVN. Later at E16.5, these catecholaminergic biosynthetic enzymes are also enriched in the bundle of His fibres, and in the interventricular septum where Purkinje fibres would later develop [17]. Both of these occur prior to cardiac innervation by sympathetic neurons [17,28], and so are likely to arise from ICA contributions. Additionally, Pnmt+ NCDs appear to contribute to the development of the heart's conduction system, as they can be readily located in association with fibres from the bundle of His [4] and Purkinje fibres [55], and ablation studies of neural crest cells suggest abnormalities in the development of the conduction fibres of the heart without neural crest invasion [56]. However, it remains unsure exactly what role, if any, that catecholamines play in the morphological development of the conduction system, or whether other factors beyond catecholaminergic signals are associated with the results of ablation studies [57]. Additionally, the transient nature of catecholamine biosynthetic enzyme expression in the developing heart is not fully understood [17]. Evidence from cultured cells [58] and *ex vivo* hearts [59,60] appears controversial, with some evidence supporting a role for catecholamines in maintaining normal beating rhythm, but some, albeit *in vitro* evidence, suggesting that catecholaminergic signalling is not necessary for intrinsic beating rhythm [61]. Overall, catecholamines are known to be essential for cardiac development, with both tyrosine hydroxylase-deficient [62] and dopamine beta-hydroxylase-deficient mice [63,64] dying *in utero*, with disorganized and atrophied CMs, despite seemingly healthy in other organs. However, an appropriate replacement of catecholamine precusors [63], or with insertion of a human ‘rescue’ gene for tyrosine hydroxylase [65], abolished the *in utero* mortality, cementing a catecholamine-specific role in the phenotype. The mortality, in terms of identified cardiac cause, time period, and minimal non-cardiac effects, were very similar to those reported in HCN4-deficient mice [66], implicating pacemaker dysfunction as producing a similar phenotype as catecholamine dysfunction in the developing heart [31]. However, specifically adrenaline-deficient hearts with disruption of Pnmt, appear normal— suggesting a limited role of adrenaline in the developing heart. However, adrenaline might have a role in normal development and in these models perhaps alternative catecholaminergic signalling is able to compensate for the loss of adrenergic signalling [4]. Thus, while catecholamines are essential for normal cardiac development, and much evidence implicates the pacemaking/conduction system, this remains to be definitively concluded, or excluded.

### 4. Similarities of Pnmt-derived cardiomyocytes and normal cardiomyocytes

PdCMs share a number of similarities and dissimilarities with canonical CMs, as seen in table 1.

**Table 1.** Similarities and dissimilarities of PdCMs and canonical CMs.

	PdCM	canonical CMs
anatomical distribution	mostly within the left atrium and left ventricle [5,7] distributed transmurally [7]	make up all four chambers of the heart parenchyma
cellular structure	rod-shaped gross morphology [5] striated ultrastructure [5]	rod-shaped gross morphology striated ultrastructure
electrophysiology	cardiac action potential produced following initial depolarization [5] calcium transient following depolarization [5] connected to myocardial syncytium [5,67]	cardiac action potential produced following initial depolarization calcium transient following depolarization connected to myocardial syncytium
contractility	contractile response to calcium transient [5]	contractile response to calcium transient
developmental lineage	expressed Pnmt at some point in development [4] some retain Pnmt expression in adult heart [4]	lineage has never expressed Pnmt no expression of Pnmt in the adult heart

**Figure 3.**
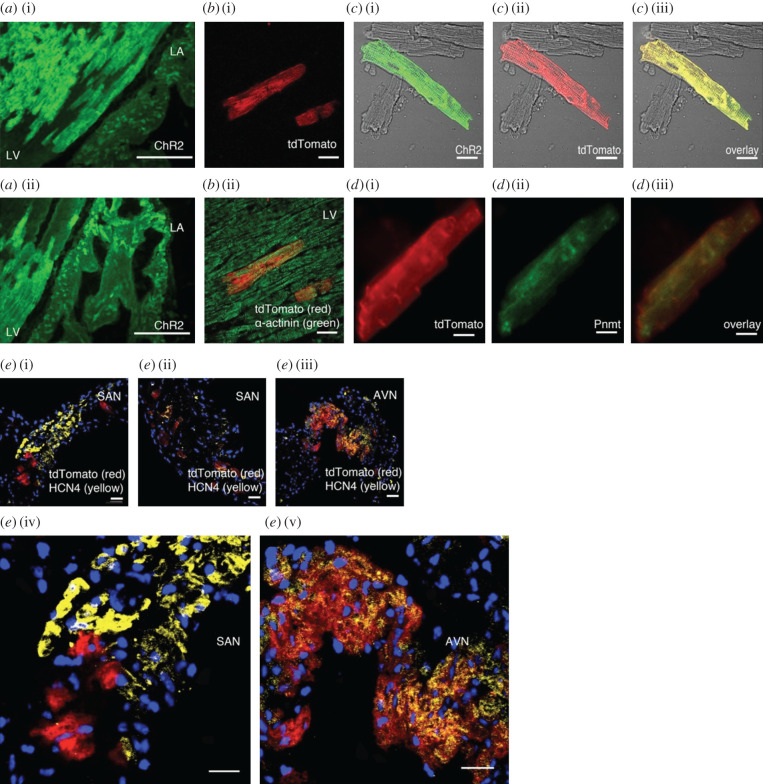
The morphology of PdCMs *in situ* in tissue and as isolated cells is indistinguishable from canonical CMs. (*a*) ChR2 staining in left ventricle (LV) and left atrium (LA) sections from adult Pnmt^Cre/ChR2^ mouse heart (*n* = 4 hearts). The majority of Pnmt-derived cells are located on the left ventricle and left atrium. (*b*) TdTomato fluorescence without (i) and with (ii) anti-α-actinin antibody staining in the left ventricle of a coronal heart section. (*c*) Immunostaining of ChR2 with anti-ChR2 antibody (i); tdTomato fluorescence in isolated LV cardiomyocytes (ii); overlay of (i) and (ii), showing co-localization of ChR2 and tdTomato (*n* = 8 cells) (iii). (*d*) Immunostaining of ChR2 with anti-ChR2 antibody (i); Pnmt staining in isolated LV cardiomyocytes (ii); overlay of (i) and (ii), showing co-expression of ChR2 and Pnmt (iii) (*n* = 4 cells). (*e*) Transverse sections from SAN (i, ii) and AVN (iii) regions stained with anti-HCN4 antibody. (iv) and (v) show the enlarged imaged of (i) and (ii), respectively. Co-localization of HCN4-tdTomato fluorescence is shown in orange. (*n* = 4 preparations). Scale bars: (*a*) 100 µm; (*b*) 20 µm; (*c*) 30 µm; (*d*) 20 µm; (*e*) 30 µm (i, ii, iii, iv and v). Figure reproduced from [5].

Morphologically, PdCMs appear indistinguishable from canonical CMs both in tissue samples and when enzymatically isolated. High magnification confocal immunofluorescence staining identifies that vital subcellular CM structures are conserved across PdCMs and canonical CMs; sarcomeric alpha-actinin readily identifies a striated subcellular distribution in PdCMs and canonical CMs. The gross cellular morphology of PdCMs identified with staining techniques is in agreement with later work using expression of a ChR2/tdTomato fusion protein in cells that have expressed Pnmt—providing an *in situ* fluorescent marker of PdCMs, without the potential for complications associated with staining techniques. ChR2/tdTomato+ PdCMs both *in situ* in cardiac tissue, and when enzymatically isolated, displayed a rectangular cellular morphology consistent with that of canonical CMs as seen in figure 3.

**Figure 4.**
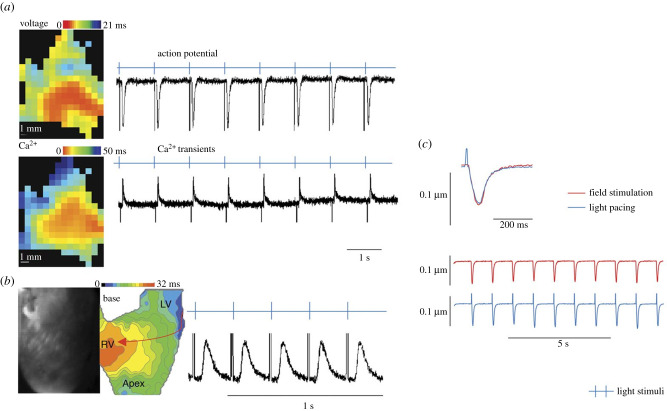
PdCMs and canonical CMs display indistinguishable electrophysiological characteristics. (a) Activation maps (left) and traces (right) of Ca^2+^ transients (upper) and action potentials (lower) obtained from a light-paced left atrium (LA) isolated from a Pnmt^Cre/ChR2^ heart and pre-incubated with Rhod-2 AM (Ca^2+^ dye) and RH237 (voltage dye). Light pulses evoked Ca^2+^ transients and action potentials in a 1 : 1 manner (*n* = 6 hearts). Light intensity: 0.6–0.8 mW mm^−2^. (*b*) Representative image (left), light pacing induced global Ca^2+^ transient activation map (middle) and fluorescence traces (right) of optical Ca^2+^ transients obtained from light paced left ventricle (LV) slices from a Pnmt^Cre/ChR2^ heart that was pre-incubated with Rhod 2-AM (Ca^2+^ dye) and RH237 (voltage dye). Light pacing resulted in an anisotropic propagation spread from LV to right ventricle (*n* = 6 hearts). Light intensity: 0.6–0.8 mW mm^−2^. (*c*) Representative sarcomere shortening (cardiomyocyte contraction) traces triggered by electric pulses (upper) and light pulses (middle) in a single PdCM isolated from a Pnmt^Cre/ChR2^ heart (*n* = 6 hearts). The lower panel shows superimposition of sarcomere shortening traces triggered by electric and light pulses. Figure reproduced from [5].

Electrophysiologically, PdCMs currently appear indistinguishable from canonical CMs, as seen in figure 4. Our novel optogenetic ChR2/tdTomato-Pnmt^Cre^ model, enabled us to selectively stimulate ChR2+ cells using pulses of 470 nm blue light [5]. We observed that following enzymatic isolation of CMs, there was a subpopulation of CMs that responded to blue light pulses, which represent ChR2+ PdCMs [5]. These cells appear to have a similar contractile response, measured by sarcomere shortening, to either field pacing or to light pacing, with light pacing specifically activating ChR2+ PdCMs [5]. Furthermore, we identified that at a tissue level [67] or in *ex vivo* whole organ preparations [5], blue light pulse activation of PdCMs reliably produced electrical membrane voltage depolarization and intracellular calcium transients, in a 1 : 1 manner, that could propagate across the myocardial syncytium [5,67]. Additionally, when targeted blue light pulses were directed at specific areas of *ex vivo* Langendorff perfused hearts, the ability to pace the heart, as measured by electrocardiogram, was only possible in the left atrium and ventricle, but not the right atrium or ventricle [5], which correlates greatly with PdCM distribution [6,7]—supporting that the blue light pulses could only pace the tissue when incident on sufficient PdCM populations to overcome some threshold of sink : source relationship with the non-optically excited canonical CMs [5,67]. Furthermore, some PdCMs are HCN4+ [4,5]. HCN4 is a channel associated with expression by pacemaker CMs in the SAN [66]. Thus, initial investigations suggest that PdCMs are similar to canonical CMs in terms of cell-surface depolarization being coupled to intracellular calcium release, and that this results in normal ECC. Additionally, PdCMs form part of the normal electrophysiologically connected myocardial syncytium, able to propagate depolarizing currents.

Thus, PdCMs appear to be grossly indistinguishable from canonical CMs in the adult heart when considering simple morphological and electrophysiological considerations [4,5]. The combination of normal sarcomeric alpha-actinin immunofluorescence staining [4,5], with normal membrane depolarization, calcium transients, and ECC [5], also suggests normal internal calcium store—membrane—myofibril junctions. These cellular similarities are in agreement with the response of whole tissue or *ex vivo* organs when PdCMs were specifically stimulated leading to similar results as with non-specific electric stimulation [4,5].

### 4.1. Dissimilarities of Pnmt-derived cardiomyocytes and normal cardiomyocytes

The very definition of PdCMs relates to their differential developmental history compared to canonical CMs. PdCMs arise from progenitors that have, at least transiently, expressed Pnmt [4,5]. However, canonical CM progenitors have not expressed Pnmt at any point [4]. These progenitors give rise to a PdCM population with a much more restricted distribution, as discussed in §2.3, when compared with canonical CMs [4–6]. However, it should be noted that the idea of a single conceptual canonical CM reflects merely the difference between those that are not derived from Pnmt+ cells, and that many subpopulations of canonical CMs do have restricted anatomical distribution—such as atrial versus ventricular [2]. Thus, the developmental history of PdCMs is dissimilar from canonical CMs, and is reflected in the restricted distribution of PdCMs.

The residual expression of Pnmt in some PdCMs appears to be the greatest difference between PdCMs and canonical CMs as first reported by S. Ebert in both the developing [4] and adult heart [6]. High magnification laser-scanning confocal fluorescent microscopy identifies that immunofluorescence for Pnmt shows strong signals in small triangular sarcomeric alpha-actinin (-) interstitial cells, presumably ICA cells, in close association around CMs [5]. However, a small number of CMs, with canonical large rod-shaped morphology, and clear striated sarcomeric alpha-actinin distribution, stain positively for Pnmt protein [5]. This has been observed both in fluorescent immunohistochemistry and immunocytochemistry [5]. However, it appears that these Pnmt+ PdCMs in the adult are a minority of the PdCM population, with limited quantitative estimates suggesting only up to 10% of PdCMs in the adult mouse left ventricle remain with significant expression of Pnmt [5]. Thus, Pnmt+ expression in CMs in the adult heart is a highly specific, but less selective, marker of PdCMs when compared with canonical CMs.

Thus, in conclusion, PdCMs and canonical CMs, with our preliminary investigations, share more cellular phenotypic similarities than dissimilarities. Electrophysiological and histological investigations suggest that both PdCMs and canonical CMs have indistinguishable electrical activity, calcium handling and conductile properties, both *in situ* [5,67], and as isolated cells [5]. However, a key differential feature is the highly specific expression of Pnmt only in adult PdCMs and not canonical CMs.

## 5. Potential roles of Pnmt-derived cardiomyocytes in the heart and future directions

### 5.1. Cellular characterization of Pnmt-derived cardiomyocytes—how similar are they to canonical cardiomyocytes?

The nature of the PdCM population is yet to be ascertained. We know only that PdCMs differ developmentally [4], in gross anatomical distribution [4,5,7], and in expression profile [5], from canonical CMs. However, as yet, beyond basic electrophysiological investigations, there has been no thorough interrogation of the phenotypic specialization of PdCMs—even with the simplest division of function in the canonical classification of CMs as working and non-working CMs [2]. It has been demonstrated in isolated ChR2/tdTomato+ cells from our Pnmt^Cre/ChR2/tdTomato^ mouse cell that optogenetic pacing induces normal electrical, intracellular signalling, and conductile properties associated with working CMs [5]. However, certain PdCMs also express HCN4 [4,5], a marker of pacemaker currents associated with non-working CMs [66]. Perhaps PdCMs populations themselves are composed of subpopulations of working and non-working PdCMs. Thus, despite our basic investigation of PdCMs, there remains to be evidence that definitively classifies PdCMs into the current simple classification of CMs.

While basic electrophysiological parameters appear indistinguishable between PdCMs and canonical CMs [5], further characterization is needed. While studies of isolated PdCMs, and PdCMs *in situ* suggest normal electrophysiological properties, including normal excitation-contraction coupling [5]; it is important to recognize that there might be differences in other electrophysiological parameters, such as the response to cellular stresses, such as adrenergic and mechanical stressors. Furthermore, identifying how excitation-transcription coupling might differ between PdCMs and canonical CMs, is an important factor to consider—as this might differ greatly and would putatively affect the response to a range of different signals.

### 5.2. Pnmt-derived cardiomyocytes in adrenergic control of the heart—do they contribute?

PdCMs might contribute to the adrenergic regulation of the heart [68]. The heart is innervated by sympathetic neurons, nearly all arising from the stellate ganglion [69,70]. These sympathetic neurons innervate both the atria and ventricles [71,72], and regulate chronotropty, dromotropy, lusitropy and inotropy of the myocardium [73,74]. These neurons appear to exist in distinct subpopulations—with larger diameter neurons expressing all the catecholamine biosynthetic enzymes, storing catecholamines, primarily noradrenaline, in vesicles in varicosities [75]. The neurons release the catecholamines en passant [76], bathing the local environment non-specifically. However, more specific interneuronal interactions do occur in connecting these sympathetic neurons to the rest of the intrinsic cardiac nervous system [77].

#### 5.2.1. The relationship between Pnmt-derived cardiomyocytes and intrinsic cardio adrenergic cells

For a long time, it was believed that this neuronal innervation was the only source of local catecholamines in the heart. However, work by Huang *et al.* in the late 1990s used a range of Northern and Western blots to demonstrate a non-neuronal source of catecholamine biosynthetic enzymes in the heart [28]. Furthermore, these were identified by electron microscopy to be rich in vesicles for secretion, and co-culture of these cells with neonatal myocytes increased basal beating rate in a beta-adrenoreceptor dependent manner [28]. Since then ICAs have remained an area of active research, with implications both in cardiac physiology [18] and pathophysiology [78], although overall ICAs are still poorly understood. However, it has been proposed that adult Pnmt+ PdCMs probably share some functional similarities to ICAs [70]. Given a likely shared developmental history, and both ICAs and PdCMs being distributed among canonical CMs [4,5], there is some logic *a priori* that PdCMs and ICAs might contribute to the adrenergic regulation of the heart, alongside sympathetic neurons [70]. CMs are also known to have some secretory mechanisms, such as of the natriuretic peptides [79]. However, whether the secretory machinery of PdCMs is sufficient to support the storage and release of catecholamines in a way similar to ICAs remains unestablished [18,28]. Thus despite their shared developmental history, it remains unclear whether PdCMs possess the secretory phenotype of ICAs. Recently, there were reports on previously undescribed intimate interactions between sympathetic neurons and local specialized cell types in adipose tissue [80,81]. It is fairly uncontroversial to suggest that ICAs and PdCMs might have evolved as local adrenergic signal modulators in a similar manner as sympathetic neuron-associated macrophages in adipose tissue [81]. However, whereas ICAs in the adult heart express all catecholamine biosynthetic enzymes to produce the terminal metabolite—adrenaline [28]; PdCMs do not express the early enzymes of tyrosine hydroxylase, nor dopamine hydroxylase, even if they do express Pnmt [70]. Thus, PdCMs appear unable to synthesize catecholamines de novo, but would putatively have to interact with their microenvironment to uptake noradrenaline, putatively from ICAs or sympathetic neurons, in order to synthesize adrenaline [70]. In the healthy heart, most noradrenaline is taken back up into sympathetic neurons via the noradrenaline transporter (NET). However, additional mechanisms for non-neuronal uptake of catecholamines exist—such as that mediated by transporter protein 3 (EMT/OCT3) [22]. If PdCMs possess catecholamine uptake mechanisms, this would expand greatly on the role that PdCMs in the adult could contribute to the local fine-tuning of the adrenergic system, and could be either through uptake, conversion to adrenaline, and subsequent release, perhaps emphasizing the receptor affinity differences between noradrenaline and adrenaline [82]. Otherwise, the uptake and degradation of catecholamines could contribute to the regulation and termination of catecholaminergic signalling locally as reported in adipose tissue [81]. The involvement of CMs in a wider neuronal-led regulatory system in the heart has already conceptually been identified [83,84]. The parasympathetic nervous system, coordinates with the sympathetic nervous system in a ‘mutually antagonistic’ manner in the heart, primarily in the atria, to effect a negative chronotropic and dromotropic effect [77,85]. However, it was identified that CMs express all the synthetic machinery for the production, storage, and release of ACh [14,84], which is the main parasympathetic neurotransmitter. Furthermore, this CM-specific ACh appears to be functionally relevant in maintenance of normal CM calcium handling, and when disrupted, such as in CM-specific vesicular ACh transporter deficient mice that are unable to store ACh in vesicles for release, maladaptive cardiac remodelling and associated haemodynamic impairment ensue [15,16]. Furthermore, it has been suggested that these ACh-producing CMs might act to amplify the ACh signal from the parasympathetic neurons [86]. It is tempting to suggest that Pnmt+ PdCMs might occupy a similar role as ACh-producing CMs, especially given the overlap of PdCM-rich areas of the heart with densely sympathetically innervated areas of the heart [86]. However, ACh is a simple molecular neurotransmitter to synthesize, using readily available biosynthetic precursors that are present irrespective of a specific cellular transcriptional programme. Thus, perhaps this increased complexity in the production of catecholamines mandates a different relationship than for ACh-producing CMs; where PdCMs are much more reliant on the catecholamine precursor supply from other non-PdCM cellular sources. Perhaps future study could focus on resolving the putative interactions between PdCMs, ICAs, and sympathetic neurons—examining the potential for multicellular flux of catecholamine molecules, such as through secretion and subsequent uptake via cell-surface membrane transporters. Further characterization of the cellular secretory phenotype, if any, of PdCMs would provide a significant development to the field of adrenergic regulation of the heart.

#### 5.2.2. Pnmt-derived cardiomyocytes and cardiac conduction system

Within the developing heart, PdCMs might contribute to the generation of normal rhythm and conduction fibres. Catecholamines, as discussed above in §3.3, are essential for cardiac development, and might contribute to developmental cardiac rhythm maintenance [63–65]. As discussed above in 2.3, the population of adrenergic cells within the developing heart is dynamically associated with the developing conducting and pacemaker sites in the embryonic heart [4]. For example, those that stain positive for HCN4 protein, denoting a pacemaker-like phenotype, and XGAL staining, denoting an adrenergic lineage in a Pnmt^Cre-Lacz^ model, are suggested to represent ICAs that are developing into pacemaker PdCMs within the SAN [4,31]. A role for ICAs in the developing heart remains unclear, in terms of whether their catecholaminergic phenotype contributes to patterning signals etc. [31]. However, it has been proposed that the development of ICAs into pacemaker PdCMs directly contributes to the development of the embryonic pacemaker and conduction system [4,31]. The invasion of the heart by Pnmt+ ICAs at E8.5 [4], before neural crest cell invasion at E10.5, and sympathetic innervation subsequently [17], provides a precise window during which local adrenergic signals from ICAs or PdCMs might be more important. As the ICAs undergo their putative transcriptional change from more catecholaminergic, to more myocyte-like PdCMS, it could be that PdCMs continue to contribute to some adrenergic signalling locally, while concomitantly developing their myocyte-like expression profile [70]. However, that Pnmt deficient mice appear to have normal gross phenotypes at rest [4] does not support this. Further investigation of the conduction pathways, including under stress [4], in these Pnmt deficient mice is required to ascertain whether the adrenergic deficiency has led to a more subtle pacemaking/conduction phenotype. Furthermore, using novel high-resolution cellular techniques such as single-cell RNA sequencing, on developing PdCM populations, might provide further clarity on the potential roles of PdCMs in the developing and early heart. An understanding of whether PdCMs contribute to the developing heart beyond simply providing CMs, might elucidate further understanding of the role of adrenergic signalling in the patterning of the developing heart; an understanding of this underlies our study of congenital heart defects, which arise in 1.35 million newborns each year [87]—representing a common heart problem that mandates further study of the contributions of PdCMs.

#### 5.2.3. Pnmt-derived cardiomyocytes and cardiac pathology

PdCMs might contribute to cardiac pathology both acutely and chronically. Catecholamines can be released in great quantities within the heart during certain acute stresses, such as myocardial ischaemia [88,89] and infarct [90]. The release of catecholamines increases myocardial oxygen demand [91], exacerbates heterogeneity in underlying conduction pathways [92,93], and can lead to CM calcium handling dysregulation [54,94]—alongside other mechanisms, these underlie the contribution of excessive catecholamines to precipitating potentially fatal cardiac dysrhythmias [51,94,95]. Furthermore, excessive catecholaminergic activity is associated with several chronic cardiac pathologies such as heart failure [96–98], stress-induced cardiomyopathy [99,100] and diabetic cardiomyopathy [101]. The mechanisms underlying the contributions of catecholamines to the cardiovascular pathophysiology of these conditions are complex and active areas of research beyond the scope of this chapter. Nonetheless, the involvement of catecholamines does suggest that if PdCMs do contribute to the regulation of local catecholaminergic signalling, then their involvement in these cardiac pathologies warrants investigation. Even if PdCMs do not have any adult adrenergic signalling capability, perhaps PdCMs can dedifferentiate to a more adrenergic phenotype in disease states. CM fetal-like phenotypes are increasingly reported in disease states such as heart failure's reversion to a fetal-like metabolic state [102]. Perhaps the integration of such fetal, or perhaps developmental-like CM cellular phenotypes might lead to PdCMs reverting to a more ICA-like phenotype [70]. Additionally, evidence supports the role of glucocorticoid-mediated regulation of cardiac Pnmt expression [103–105], some of which might be mediated by effects on PdCMs. One particular stress-induced cardiomyopathy—Takotsubo cardiomyopathy presents with hypertrophy and remodelling primarily at the base of the heart [99,100]. Others have attempted to suggest apico-basal variations in the response to catecholamines, such as through differential subcellular cAMP domain regulation [12]. However, given the selective distribution of PdCMs, with significant distribution around the base of the heart [5–7]; it is tempting to speculate Takotsubo cardiomyopathy might be demonstrative of a particularly severe example of PdCM adrenergic dysregulation. However, within all of these suggestions, we still cannot evidence any speculation as to whether the involvement of PdCMs might be protective, or deleterious. Further study of PdCMs in disease models, such as with single-cell RNA sequencing, may help identify their disease-specific roles, and perhaps help elucidate their normal functions in healthy physiology.

PdCMs present an opportunity for exploitation experimentally. Optogenetics is a novel field that enables cell-type specific modulation and interrogation *in situ*, using light-sensitive ion channels called opsins [106]. Cardiac optogenetics is a cutting-edge application of wider optogenetic techniques [107], but is limited by the variability in viral vectors, such as AAV9, transfection efficacy in different models [108,109]. Novel approaches exploiting the developmental differences between PdCMs and canonical CMs, to express opsins in PdCMs [5], have already been used in research to produce novel methodologies that enable highly specific spatio-temporal stimulation of cardiac tissue, to a degree that was not matched by prior electrical stimulation techniques [5,67]. The identification of PdCMs as a tool for cardiac optogenetics will probably contribute to the next generation of electrophysiological discovery in the heart.

## 6. Conclusion

Our recent identification of a previously unknown differential developmental pathway for CMs has potentially far-reaching implications [4,5]. Since the first identification of PdCMs through a Pnmt^Cre-LacZ^ model [4], we have come to understand the basic similarities and dissimilarities of PdCMs and canonical CMs. Primarily, it appears that the main differences between PdCMs and canonical CMs are in the specific left-sided distribution of PdCMs in the adult heart [5–7], and that some maintain expression of Pnmt in the adult heart [5,6]. The difference in developmental history and epigenetic landscape has already been exploited for robust cardiac optogenetic study [5,67]. However, beyond this, we have much to explore. There are many ‘known unknowns’ such as whether PdCMs contribute to local adrenergic signalling in health and disease. However, it is the ‘unknown unknowns’ that we should be careful not to miss, as we stand at the intersection of novel cardiac optogenetics, cardiac autonomic regulation and fundamental cell biology.

## Data accessibility

This article has no additional data.
